# Decoding the impact of disease-causing mutations in an essential aminoacyl-tRNA synthetase

**DOI:** 10.1016/j.jbc.2021.101386

**Published:** 2021-11-06

**Authors:** Marie Sissler

**Affiliations:** ARNA - UMR5320 CNRS - U1212 INSERM, Université de Bordeaux, IECB, Pessac, France

**Keywords:** Aminoacyl-tRNA synthetase, compound-heterozygous mutations, genetic disease, multisynthetase complex, integrated stress response, aaRSs, aminoacyl-tRNA synthetases, EPRS, glutamyl-prolyl-tRNA synthetase, ERS, glutamyl-tRNA synthetase, ISR, integrated stress response, PRS, prolyl-tRNA synthetase

## Abstract

Aminoacyl-tRNA synthetases are housekeeping enzymes that catalyze the specific attachment of amino acids onto cognate tRNAs, providing building blocks for ribosomal protein synthesis. Owing to the absolutely essential nature of these enzymes, the possibility that mutations in their sequence could be the underlying cause of diseases had not been foreseen. However, we are learning of patients bearing familial mutations in aminoacyl-tRNA synthetases at an exponential rate. In a recent issue of JBC, Jin *et al*. analyzed the impact of two such mutations in the very special bifunctional human glutamyl-prolyl-tRNA synthetase and convincingly decode how these mutations elicit the integrated stress response.

Understanding the molecular basis of a disease-causing mutation and grasping the genotype–phenotype correlation could in principle be an easy task, especially when the impacted protein is an essential enzyme that catalyzes tRNA aminoacylation, a key step for mRNA translation. However, in practice, achieving such a level of understanding is far more complex. Aminoacyl-tRNA synthetases (aaRSs) are ancestral enzymes made of catalytic and anticodon-binding domains that are highly conserved among species, reflecting the essentiality of their function. Nevertheless, additional domains have been appended over the course of evolution, so that many metazoan aaRSs have become decorated with modules that allow context-dependent roles in functions unrelated to their primary and constitutive tRNA-charging activity (1). The picture is even more complex considering the fact that, in humans, numerous cytosolic aaRSs are anchored within a macromolecular complex (multisynthetase complex [MSC]), mostly stable and dedicated to improving mRNA translation efficiency by channeling charged tRNAs directly to the ribosome A site. Importantly, the MSC can additionally act as a depot for the stimulus-dependent release of regulatory proteins that then perform specialized auxiliary functions (2, 3). As elegantly pictured by Paul Schimmel, “because the (auxiliary) functions are detached from anything even remotely resembling the aminoacylation reaction, we can think of them as analogous to a caterpillar turning into a butterfly” (4). Although not the topic here, I should also note that the mitochondrial aaRSs have a similar diversity of peculiarities that renders their study just as tricky. Nevertheless, the two populations of aaRSs, cytosolic and mitochondrial, have been identified in a drastically increasing number of reports as being mutated in patients with severe disease phenotypes (5, 6).

The human glutamyl-prolyl-tRNA synthetase (EPRS) is even more special in that it is the only bifunctional enzyme with two aaRSs (glutamyl-tRNA synthetase [ERS] and prolyl-tRNA synthetase [PRS]) fused into a single polypeptide (Fig. 1). EPRS possesses in addition two extra domains: a GST-like domain that facilitates anchoring onto the MSC and a noncatalytic linker region made of three helix-turn-helix domains. Two EPRSs are required in the MSC to build one functional homodimeric (α_2_) PRS, which subsequently leads to the presence of two units of the monomeric ERS. The implication of EPRS in noncanonical functions is well documented; for instance, EPRS has a role in the γ-interferon–activated inhibition of translation system to counteract inflammation and a role in the antiviral response (reviewed in Arif *et al.* (7)). In both cases, EPRS is released from the MSC after stimulus-induced phosphorylation of residues in the linker region (Fig. 1, in blue). EPRS was first reported in 2018 as being mutated in patients with hypomyelinating leukodystrophy, a central nervous system disease (8). Interestingly, all missense variants reported at that time affected the PRS catalytic domain of EPRS (Fig. 1, in orange) in “pseudohomozygous” states. Indeed, two of the reported patients were homozygous, and the two others were compound heterozygous but with missense variants opposite nonsense alleles, which suggests that the pathogenicity relies exclusively on homodimeric mutated forms of PRS. More recently, Jin *et al*. identified new patients presenting with diabetes and bone diseases with compound-heterozygous EPRS variants (Fig. 1, in red), resulting in P14R and E205G substitutions, which are situated in the GST-like domain and the catalytic domain of ERS, respectively (9).

**Figure 1:**
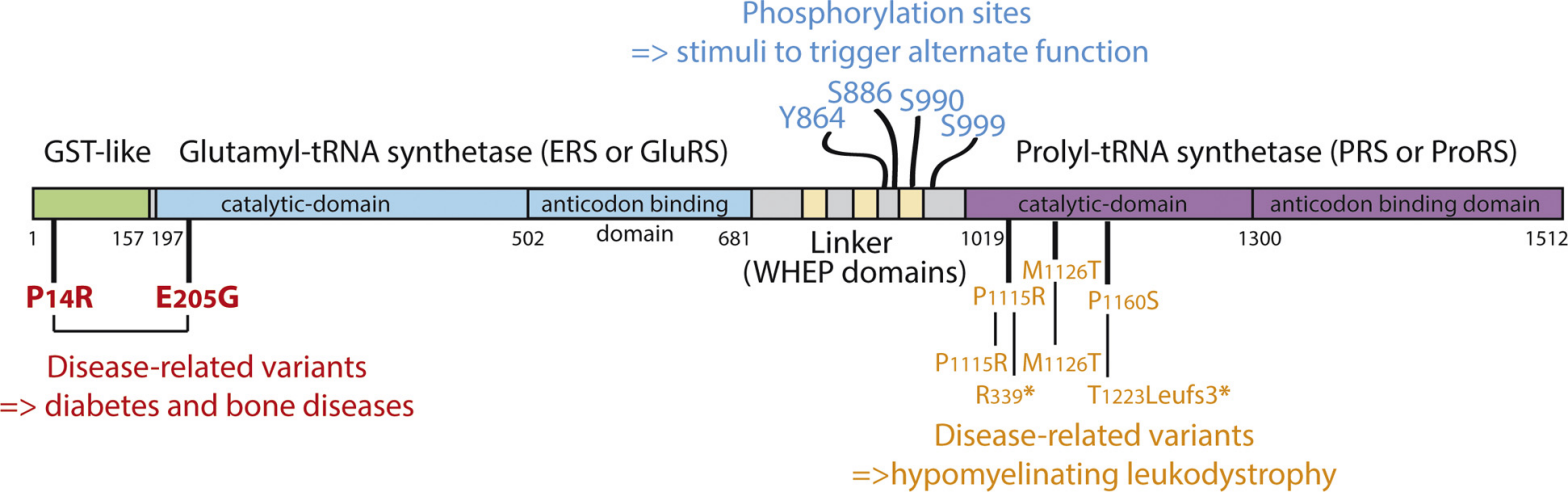
**Modular organization of the human glutamyl-prolyl-tRNA synthetase (EPRS).** The different functional domains are distinguished by color. Residues in *blue* are known to be phosphorylated under specific cellular conditions, which leads to the release of EPRS from the MSC, triggering its noncanonical function(s) (7). Missense and nonsense (∗) variants are indicated in *orange* and *red* for those reported in the studies by Mendes *et al*. (8) and Jin *et al*. (9), respectively. Allelic compositions, as identified in patients, are linked through *black lines*. MSC, multisynthetase complex.

In their study, Jin *et al*. (9) demonstrate that neither of the newly discovered missense variants altered the anchoring of EPRS into the MSC, suggesting that these mutant enzymes may perform their canonical functions. Therefore, the authors meticulously analyzed the impact of these mutations on aminoacylation. An important *in vitro* preamble to this study was to design a recombinant, soluble, and stable enzyme. The authors thus constructed a maltose-binding protein–tagged ERS, which includes part of the linker region (MBP-ERS_2.5W_), and verified that it possessed good solubility, tRNA binding, and efficient aminoacylation properties. ERS is one of the few aaRSs that requires the cognate tRNA for amino acid activation, the first step of the aminoacylation reaction. To investigate this specific step, the authors made an unloadable tRNA^Glu^ by oxidizing the terminal 3′-A76 to disable amino acid transfer. While only the E205G variant significantly impacted the aminoacylation reaction, the authors showed that the amino acid activation step was altered in both this and the P14R variant. To further characterize the impact of the P14R variant, the authors observed that the Flag-tagged P14R-EPRS overexpressed in HEK293T cells had compromised integrity, with accumulation of a ∼60-kDa truncated fragment, suggesting a potential conformational rearrangement that could render the protein more sensitive to cellular proteases. Such a long-range conformation change induced by the P14R mutation was indeed then confirmed by CD spectroscopy (which analyzes the secondary structure and thermal melting profile), making consistent the observed altered aminoacylation kinetics. Thus, both EPRS variants described in this work alter the aminoacylation of tRNA^Glu^, but do so by distinct molecular mechanisms.

The challenge, frequently raised in describing patients bearing aaRS mutations (10), is to correlate the observations of impacts on a single constitutive molecular process with phenotypes that affect some organ systems and not others. EPRS mutation–related patients described in the study by Jin *et al*. presented with diabetes and bone diseases (9). The authors wisely suggest some metabolic pathways that could be altered to lead to such symptoms, mostly related to integrated stress response (ISR). Consistently, the authors show that ISR markers, such as ATF4 and CHOP, are increased in EPRS-related patient-derived fibroblasts treated with thapsigargin, a potent inducer of endoplasmic reticulum stress. They also observe that prolonged stress contributed to the ISR reprograming of the transcriptome and was followed by a progressive loss of cell viability, providing new insights toward full comprehension of the disease phenotypes.

The difficulty in understanding diseases linked to aaRSs relies on the essentiality of the enzyme's function(s), which cannot be too drastically impaired (otherwise it is often compensated for in the heterozygous composition by a variant with less-severe consequences). Understanding the molecular impact(s) that lead(s) to disease requires meticulous study because it often consists of identifying subtle, discrete, and probably cumulative molecular effects. It further relies on the fact that most of the aaRSs have several functions and thus distinct sets of molecular partners specific to each of these functions. It cannot be ignored that each of these processes and partnerships may be impacted by the disease-causing variants, rendering essential a comprehensive fundamental knowledge of these enzymes. Thanks to their work, Jin *et al*. have established an excellent experimental setup to investigate patient-related ERS variants, which will be of use in future clinical diagnoses, which are becoming increasingly more likely to occur in a context where new descriptions of patients emerge exponentially.

## References

[1] Guo M., Schimmel P. (2013). Essential nontranslational functions of tRNA synthetases. Nat. Chem. Biol..

[2] Ray P.S., Arif A., Fox P.L. (2007). Macromolecular complexes as depots for releasable regulatory proteins. Trends Biochem. Sci..

[3] Hyeon D.Y., Kim J.H., Ahn T.J., Cho Y., Hwang D., Kim S. (2019). Evolution of the multi-tRNA synthetase complex and its role in cancer. J. Biol. Chem..

[4] Schimmel P. (2020). The endless frontier of tRNA synthetases. Enzymes.

[5] Jiang L., Jones J., Yang X.-L. (2020). Human diseases linked to cytoplasmic aminoacyl-tRNA synthetases. Enzymes.

[6] González-Serrano L.E., Chihade J.W., Sissler M. (2019). When a common biological role does not imply common disease outcomes: Disparate pathology linked to human mitochondrial aminoacyl-tRNA synthetases. J. Biol. Chem..

[7] Arif A., Yao P., Terenzi F., Jia J., Ray P.S., Fox P.L. (2018). The GAIT translational control system. Wiley Interdiscip. Rev. RNA.

[8] Mendes M.I., Gutierrez Salazar M., Guerrero K., Thiffault I., Salomons G.S., Gauquelin L., Tran L.T., Forget D., Gauthier M.S., Waisfisz Q., Smith D.E.C., Simons C., van der Knaap M.S., Marquardt I., Lemes A. (2018). Bi-allelic mutations in EPRS, encoding the glutamyl-prolyl-aminoacyl-tRNA synthetase, cause a hypomyelinating leukodystrophy. Am. J. Hum. Genet..

[9] Jin D., Wek S.A., Kudlapur N.T., Cantara W.A., Bakhtina M., Wek R.C., Musier-Forsyth K. (2021). Disease-associated mutations in a bifunctional aminoacyl-tRNA synthetase gene elicit the integrated stress response. J. Biol. Chem..

[10] Kuo M.E., Antonellis A. (2020). Ubiquitously expressed proteins and restricted phenotypes: Exploring cell-specific sensitivities to impaired tRNA charging. Trends Genet..

